# Mediation of *Drosophila *autosomal dosage effects and compensation by network interactions

**DOI:** 10.1186/gb-2012-13-4-r28

**Published:** 2012-04-24

**Authors:** John H Malone, Dong-Yeon Cho, Nicolas R Mattiuzzo, Carlo G Artieri, Lichun Jiang, Ryan K Dale, Harold E Smith, Jennifer McDaniel, Sarah Munro, Marc Salit, Justen Andrews, Teresa M Przytycka, Brian Oliver

**Affiliations:** 1 Laboratory of Cellular and Developmental Biology, National Institute of Diabetes and Digestive and Kidney Diseases, 50 South Drive, Bethesda, MD 20892, USA; 2 Department of Biology, Florida State University, 319 Stadium Drive, Tallahassee, FL 32306, USA; 3 Computational Biology Branch, National Center for Biotechnology Information, National Library of Medicine, National Institutes of Health, 8600 Rockville Pike, Bethesda, MD 20814, USA; 4 Department of Biology, 385 Serra Mall, Stanford University, Stanford, CA 94304, USA; 5 Human Genome Sequencing Center, Baylor College of Medicine, 1 Baylor Plaza, Houston, TX 77030, USA; 6 Genomics Core, National Institute of Diabetes and Digestive and Kidney Diseases, 8 Center Drive, Bethesda, MD 20814, USA; 7 Biochemical Science Division, Molecular Measurement Laboratory, National Institute of Standards and Technology, 100 Bureau Drive, Gaithersburg, MD 20899, USA; 8 Department of Biology, Indiana University, 1001 East 3rd Street, Bloomington, IN 47405, USA

## Abstract

**Background:**

Gene dosage change is a mild perturbation that is a valuable tool for pathway reconstruction in *Drosophila*. While it is often assumed that reducing gene dose by half leads to two-fold less expression, there is partial autosomal dosage compensation in *Drosophila*, which may be mediated by feedback or buffering in expression networks.

**Results:**

We profiled expression in engineered flies where gene dose was reduced from two to one. While expression of most one-dose genes was reduced, the gene-specific dose responses were heterogeneous. Expression of two-dose genes that are first-degree neighbors of one-dose genes in novel network models also changed, and the directionality of change depended on the response of one-dose genes.

**Conclusions:**

Our data indicate that expression perturbation propagates in network space. Autosomal compensation, or the lack thereof, is a gene-specific response, largely mediated by interactions with the rest of the transcriptome.

## Background

Systematic evaluation of gene dose in segmental aneuploids shows that dose changes in the majority of the *Drosophila *genome are compatible with life [1,2], but if there are enough changes in dose, regardless of the particular genes, viability is greatly reduced [2]. This suggests that gene dose changes have small additive effects on viability in *Drosophila*, which may be analogous to the situation in humans, where small regions of segmental aneuploidy are associated with subtle adult phenotyes (for example, disease) and large departures from ploidy result in fetal death [3-5]. The small effect of gene dose, and the significant additive effects when there are enough of these changes, suggest that large departures from gene balance collapse genetic networks. Understanding the effect of copy number on gene expression is a prerequisite for systematic study of gene dose as a network attribute.

While there are clear dose effects on viability in segmental aneuploids [2] and in dominant genetic interactions in *Drosophila *(for example, [6]), the effect of copy number may be less than implied by the gene dose *per se*. One-dose genes in flies heterozygous for deficiencies (deletions removing multiple loci) show average expression values less than two-fold reduced [7-9]. Expression also shows a sublinear relationship to gene dose in highly aneuploid *Drosophila *tissue culture cells [10]. In whole *Drosophila *showing aneuploidy, some genes in trisomic regions show compensation, while others do not, at both the transcript and protein levels [11,12]. All these data indicate that gene dose responses are not always a simple reflection of copy number. We do not have well-developed models for the important relationship between gene dose and expression in *Drosophila*, but there are at least two general mechanisms that we test here.

One model for autosomal dosage compensation suggests that deletions in autosomes are recognized as aneuploid segments and partially compensated in a fixed-fold manner independent of the specific gene. There is strong evidence that extensive chromosome-level aneuploidy results in a characteristic stress response in both yeast and mouse cells [13]. One can imagine, therefore, that a compensatory response to aneuploidy would be advantageous for cells (although perhaps not organisms, which might rather purge aneuploid cells). There is good agreement in average autosomal dosage compensation levels reported in *Drosophila *[8,9,14], which would be expected if a global aneuploid recognition/correction system existed. Indeed, there are at least two such systems. Wild-type *Drosophila *are diploid for two major autosomes, a dot autosome (chromosome 4), and have either one (males) or two × chromosomes (females). Much of the work on the gene dosage in *Drosophila *has focused on the × chromosome, where a chromatin-remodeling machine (the male-specific lethal (MSL) complex) recognizes and decorates the × to increase gene expression in males [15] by promoting transcriptional elongation [16]. However, the small fourth chromosome is also recognized by a chromatin remodeling machine (Painting of fourth, Pof) to increase gene expression [7]. An analogous global mechanism could partially and uniformly compensate for segmental aneuploid regions that arise by mutation on the remaining two major autosomes. If such a system exists, then expression of a common set of genes encoding this machinery would be expected to increase in segmental aneuploid *Drosophila*, regardless of the particular location of the aneuploid segment. Even in the case of these remodeling systems the relationship between dose and expression is not simple. There is an × chromosome-specific compensation system in *Drosophila *that acts in the soma during embryogenesis, but not in the germline. Some genes on the × chromosome in males show dosage compensation prior to the activation of the principal dosage compensation system in the soma [17], and × chromosome genes in the mitotic male germline, where the somatic dosage compensation system does not operate, are tightly dosage compensated [9,18].

There are other possible dosage compensation mechanisms. A second model suggests that feedback mechanisms [19] and the dampening of dose effects due to the kinetic properties of flux through networks [20] result in partial dosage compensation depending on the specific gene with a dose change. This network model is supported by the fact that gene dose manipulation is a powerful pathway reconstruction tool in *Drosophila*, where deficiencies result in a sensitized genetic background for discovering new pathway members [21,22]. These studies strongly suggest that gene dose reductions for individual loci result in reduced gene activity and a subtle propagation of perturbations into regulatory networks. In classical genetic terms, this suggests that many genes may have subliminal haplo-insufficient properties, resulting in synthetic or background-dependent phenotypes only when nearby gene activities in the pathway are suboptimal. This gene-specific response hypothesis makes three clear predictions: 1) genes should show individual characteristic expression responses to reduction in dose; 2) these responses should occur in the context of the gene expression network in which they are embedded; and 3) expression deviations from genes with reduced dose should propagate into the expression network.

Our work on gene expression in a series of *Drosophila *deficiencies analyzed in the context of different network models indicates that gene interactions play a large role in autosomal dose effects and dosage compensation. We suggest that studies in model organisms, with a more controlled genetic background and environment, will help us parse out the complexities of gene dose effects and interactions among large sets of genes that make small contributions to overt morphological or physiological phenotypes in development and disease.

## Results

### Drosophila lines with reduced gene dose

**Figure 1:**
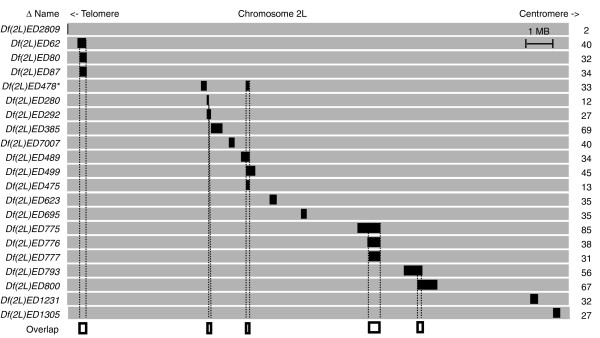
**Lines used**. Positional map of the start and stop positions (black bars) for deficiencies (*Df(2L)EDs*, on left) profiled along chromosome 2L (gray bars) and the number of coding genes (on right) removed in full or part. Each line had a single deficiency region with the exception of one line (asterisk) containing *Df(2L)ED478 *as well as the *de novo Df(2L)Hsp60c*. Overlapping deficient regions are shown (dashed lines, open bars).

We took advantage of the *Drosophila *model system by measuring genome-wide mRNA expression in engineered autosomal deficiency lines (*Df*/*+*) from the European *Drosophila *deletion collection (DrosDel) project [22,23]. The DrosDel collection offered a key experimental advantage, as all strains are from the same original stock, minimizing genetic background outside of the engineered deletion. We selected 21 DrosDel deficiency lines from chromosome arm 2L to survey mRNA responses to gene dose in adult flies (Figure 1). The test set allowed us to look at one-dose genes in five regions with multiple deficiencies, so that we could explore the question of whether compensation is a property of individual genes or particular deficiencies. The *Dfs *removed a variable number of genes and were scattered along the length of the chromosome arm.

While the engineered deletions we used have molecularly defined breakpoints, spontaneous rearrangements do occur. Additionally, *Drosophila *has many tissues with variably endoreplicated genomes [24], which might provide a corrective amplification. To directly assess gene dose in the *Df*/*+ *flies, we performed DNA-sequencing (DNA-Seq, average sequencing depth 3.2×) on adult females and males of all 21 lines and the parental line. We aligned genome-wide to confirm genotypes (Table 1; Additional file 1), to test for selective endoreplication, and to detect any novel structural rearrangements. While we did observe known selective endoreplication events at the chorion loci in females, we found no evidence to support the idea that selective amplification was part of the dosage compensation response. DNA-Seq coverage of wild-type autosomes was two-fold higher (standard deviation (SD) = 0.2) than in the engineered deletion regions, indicating that all *Df*s reduce gene dose by half. In one case (*Df(2L)ED478*), we found an additional uncharacterized deficiency, which we named *Df(2L)EDHsp60c*, but otherwise we detected no overt novel rearrangements elsewhere in the genomes, indicating that dose had not deviated in the time following creation of the original engineered deletions.

**Table 1:** DNA-Seq measurements for aneuploid segments

Deficiency	First missing base	Last missing base	Measured in	RPM in *Df *region	RPM in WT	Fold difference
*Df(2L)ED2809*/*+*	67365	72671	Female	29.5	54.3	1.84
*Df(2L)ED2809*/*+*	67365	72671	Male	27.9	60.3	2.16
*Df(2L)ED62*/*+ *	480873	826788	Female	1,371.5	2,912.8	2.12
*Df(2L)ED62*/*+ *	480873	826788	Male	1,366.1	3,313.8	2.43
*Df(2L)ED80*/*+*	568095	850645	Female	1,168.28	2,373.4	2.03
*Df(2L)ED80*/*+*	568095	850645	Male	1,253.1	2,691.5	2.14
*Df(2L)ED87*/*+ *	568095	852827	Female	1,103.5	2,390.5	2.17
*Df(2L)ED87*/*+ *	568095	852827	Male	1,177.3	2,711.5	2.30
*Df(2L)EDHsp60c*/*+ *	5564618	5809650	Female	1,060.5	2,151.0	2.03
*Df(2L)EDHsp60c*/*+ *	5564618	5809650	Male	1,170.1	2,362.2	2.02
*Df(2L)ED280*/*+ *	5801930	5907456	Female	447.8	922.5	2.06
*Df(2L)ED280*/*+ *	5801930	5907456	Male	509.2	970.4	1.92
*Df(2L)ED292*/*+*	5801930	5981009	Female	744.8	1,510.1	2.03
*Df(2L)ED292*/*+*	5801930	5981009	Male	804.1	1,648.5	2.05
*Df(2L)ED385*/*+ *	5980272	6465772	Female	1,801.7	3,841.6	2.13
*Df(2L)ED385*/*+ *	5980272	6465772	Male	2,130.8	4,350.7	2.04
*Df(2L)ED7007*/*+*	6709099	6963808	Female	1,989.6	2,107.4	1.06
*Df(2L)ED7007*/*+*	6709099	6963808	Male	1,401.4	2,308.7	1.65
*Df(2L)ED489*/*+*	7204186	7576637	Female	1,692.6	3,338.0	1.97
*Df(2L)ED489*/*+*	7204186	7576637	Male	1,803.2	3,485.6	1.93
*Df(2L)ED499*/*+ *	7423765	7800182	Female	1,727.3	3,337.2	1.93
*Df(2L)ED499*/*+ *	7423765	7800182	Male	1,844.5	3,514.0	1.91
*Df(2L)ED475*/*+ *	7423915	7576637	Female	700.2	1,428.4	2.04
*Df(2L)ED475*/*+ *	7423915	7576637	Male	786.9	1,474.1	1.87
*Df(2L)ED478*/*+ *	7437442	7576637	Female	581.2	1,309.2	2.26
*Df(2L)ED478*/*+ *	7437442	7576637	Male	628.7	1,348.3	2.14
*Df(2L)ED623*/*+ *	8403564	8700124	Female	1,335.1	2,531.3	1.90
*Df(2L)ED623*/*+ *	8403564	8700124	Male	1,504.1	2,718.2	1.81
*Df(2L)ED695*/*+*	9699225	9918192	Female	929.1	1,858.3	2.00
*Df(2L)ED695*/*+*	9699225	9918192	Male	1,009.2	2,041.9	2.02
*Df(2L)ED775*/*+ *	12010010	12975028	Female	4,424.3	7,821.9	1.77
*Df(2L)ED775*/*+ *	12010010	12975028	Male	4,758.8	8,352.9	1.76
*Df(2L)ED776*/*+ *	12434538	12975028	Female	2,086.6	4,337.0	2.08
*Df(2L)ED776*/*+ *	12434538	12975028	Male	2,347.3	4,638.7	1.98
*Df(2L)ED777*/*+ *	12484452	12975028	Female	1,997.0	3,910.5	1.96
*Df(2L)ED777*/*+ *	12484452	12975028	Male	2,387.6	4,182.3	1.75
*Df(2L)ED793*/*+*	13934848	14689337	Female	3,324.0	6,563.4	1.97
*Df(2L)ED793*/*+*	13934848	14689337	Male	3,619.5	7,125.3	1.97
*Df(2L)ED800*/*+*	14490575	15332688	Female	3,750.7	6,968.0	1.86
*Df(2L)ED800*/*+*	14490575	15332688	Male	4,032.9	7,456.4	1.85
*Df(2L)ED1231*/*+ *	19158440	19464056	Female	1,266.4	2,533.5	2.00
*Df(2L)ED1231*/*+ *	19158440	19464056	Male	1,362.8	2,789.8	2.05
*Df(2L)ED1305*/*+ *	20085397	20382385	Female	932.4	1,831.8	1.96
*Df(2L)ED1305*/*+ *	20085397	20382385	Male	1,003.9	2,005.4	2.00
Mean						1.97
Standard deviation						0.21

WT, wild type.

We performed expression experiments on both females and males because, in a network model for dose effects and compensation, the responses to gene dose should differ depending on expression context. There is a long history of expression profiling between the sexes that has clearly shown that females and males have substantially different expression networks, due in large measure to the gonads and particularly the germ cells [9,25-28]. Because of the large gonad size relative to the rest of the body, these sex-biased expression profiles are quite evident in whole adults. While there are advantages to examining expression networks by cell type, tissue or organ, we were concerned about introducing dissection as a variable in the experiments; therefore, we performed all work on whole females and males.

### Expression of one-dose genes in *Df*/+ flies

**Figure 2:**
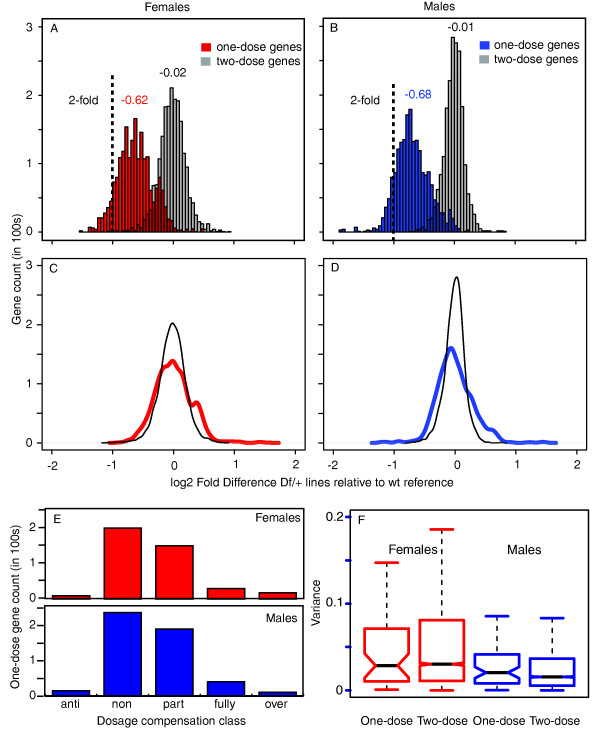
**Expression of one-dose genes**. (a,b) Histograms of expression values for one-dose genes and the same number of two-dose genes generated by resampling expression values (2,000×). This corrects for the large sample size differences between these two dose classes. Resampling of two-dose genes was restricted to contiguous regions corresponding in gene content to the extent of gene deletion in *Df*s to control for nonrandom expression values resulting from co-regulation of physically linked genes. The expected value for non-compensation is shown (dotted line). Mean expression differences are indicated above each distribution. (c,d) Mean centered distributions of the graphs in (a,b). (e) Prevalence of dosage compensation classes (see Materials and methods). (f) Notched boxplots of variance (fold-difference^2^) calculated for sampled (2,000×) one-dose genes due to different *Df*s compared to variance of the same genes in a two-dose state. Medians (bar), 95% confidence intervals (notch), 25 to 75 percentiles (box), and 1.5 × interquartile range (whiskers) are shown. Wt, wild type.

To determine the overall pattern of dose responses, we pooled expression measurements for all 478 one-dose genes in the entire set of deficiencies and compared expression to a wild-type reference built from the same set of experiments (Additional file 2). We then used resampling methods to compare the expression of similar numbers of one-dose and two-dose genes. Because expression of genes physically linked on chromosomes are often correlated [29], we sampled contiguous blocks of two-dose genes along the genome to obviate any effects due to the non-random arrangement of genes. As expected, we observed lower expression from the one-dose genes (Figure 2a,b). Females and males showed similar overall responses to this copy number change with a mean 1.6-fold reduction in expression. This was less expression change than the two-fold reduction predicted if expression strictly followed gene dose, and is in line with previous studies [7].

The distribution of responses around means could be due to biological and technical noise layered over a 1.6-fold partial-compensation system that the cellular machinery applied uniformly to all one-copy genes, or gene-specific responses due to feedback. If there were a general fixed-fold aneuploid response system, then error and noise should be normally distributed around the central tendency of 1.6-fold expression compensation. This was not the case. Mean centered distributions showed extended tails and skewing towards better dosage compensation when compared to the expression distributions of two-dose genes (Figure 2c,d). The distribution of one-dose genes was not normal (*P *< 0.01, Jarque-Bera test), and the differences in distribution shape of one-dose and two-dose genes was significant (*P *< 0.01, Kolmogorov-Smirnov test), indicating that the spread in the expression values of one-dose genes was not due to measurement error or biological noise in the system. The response to reduced dose was heterogeneous in nature.

For analyses we will present later in the manuscript, it was useful to classify the dosage compensation responses. Genes within the bounds of models for fully or non-compensated were classified as such. We classified genes failing both models (*P *< 0.05) as anti-, partially or over-compensated based on the position relative to the two models (see Materials and methods). Non-compensation and partial compensation classes accounted for the most genes, but we also observed skewing toward better compensation following classification (Figure 2e). These classifications also show the heterogeneous nature of the dose response, and suggest that dosage compensation responses were gene-specific.

To more directly test for a gene-specific response, we asked if the dosage compensation response of a given one-dose gene was significantly different when tested in the context of different deficiencies, which all uncover the same one-dose gene. This test had the added advantage of exploring the idea that there are deletion-specific compensation levels. Such effects might be mediated by changes in the complex three-dimensional structure of the nucleus arising from deletions and juxtaposition of breakpoints. We observed no significant differences in expression when the same one-dose gene was measured in the context of different deficiencies. Additionally, we analyzed variance in expression among one-dose genes compared to those same loci when present in two doses. There was no significant change in expression variance due to gene dose (Figure 2f). We also found no significant correlation between *Df *extent (amount of DNA or number of contiguous genes removed), or position along the chromosome, and compensation class (Additional file 3). These data indicate that the one-dose response was gene-specific.

**Figure 3:**
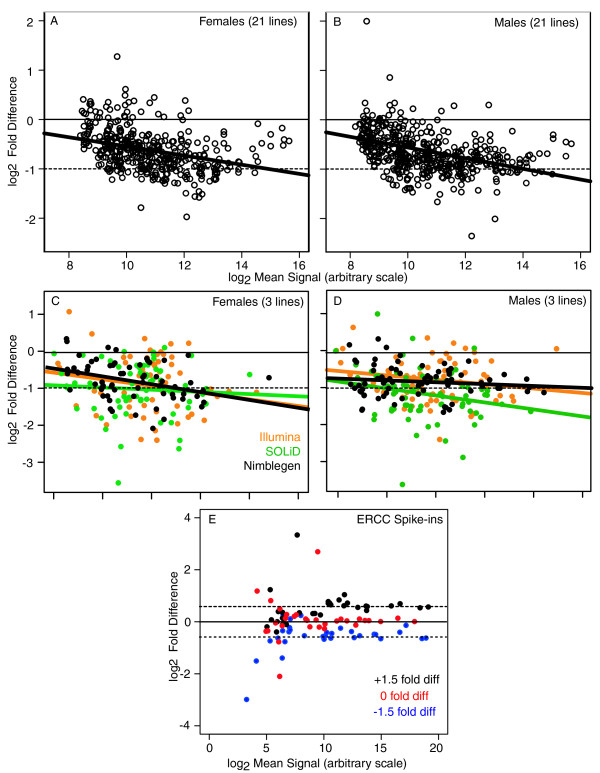
**Dosage compensation and expression level**. **(a-d) **Ratio intensity (MA) scatterplots showing two-dose array expression values plotted against fold difference in expression in one-dose versus two-dose. (a,b) The full 21-line data set from microarray experiments. Expected values for full compensation (thin solid lines) and non-compensation (thin dashed lines) are shown. (c,d) Data from three lines tested by Illumina RNA-Seq (yellow), SOLiD RNA-Seq (green) or Nimblegen array (black). Trend-lines for each plot are shown (bold lines). (e) Measurements on External RNA Control Consortium (ERCC) control RNAs of known relative abundance (for example, spike-ins) with 1:1 and 1.5:1 ratios across the two mixtures (mix 1 and mix 2). (c,d,e) Expected values for 1:1 (solid line) and the two 1:1.5 ratios (dotted lines) are shown.

We found a clear correlation (*P *< 0.01) between expression level and compensation state. No compensation was more prevalent at high expression levels, while compensation was more prevalent at lower expression levels (Figure 3a,b). However, it is important to note that we observed a range of reproducible responses to dose at all expression levels. One has to be particularly careful with assessing compensation levels in expression experiments, as technical noise at low expression levels can falsely suggest compensation. We were rigorous with low-level cutoff values (>2 SD above background in all 21 lines; see Materials and methods), strongly suggesting that better observed compensation at low expression levels was not due to spurious ratiometric values due to noise. Importantly, we found support for the array data by Illumina and SOLiD RNA-Seq on three of the deficiency lines, suggesting that genes with lower expression levels were indeed more fully compensated (Figure 3c,d). Furthermore, we used sets of 96 control RNAs of known abundance as external RNA spike-ins produced by the External RNA Control Consortium (ERCC) [30] to calibrate expression ratios over a range of expression values in these experiments. Data from the SOLiD platform showed linearity between the observed and expected abundances and lack of data compression (Figure 3e). We have previously demonstrated the linearity of Illumina RNA-Seq with these controls [31] as well as linearity between RNA-Seq and arrays [32]. We concluded that the negative relationship between compensation and expression levels was a biological phenomenon.

Gene regulation might explain compensation responses of individual genes. For example, genes showing anti-compensation could be auto-regulatory and the loss of one copy might create a downward spiral due to loss of positive feedback. It follows that gene-specific dosage compensation mediated by network interactions should change as the structure of the network and associated gene expression levels changed.

**Figure 4:**
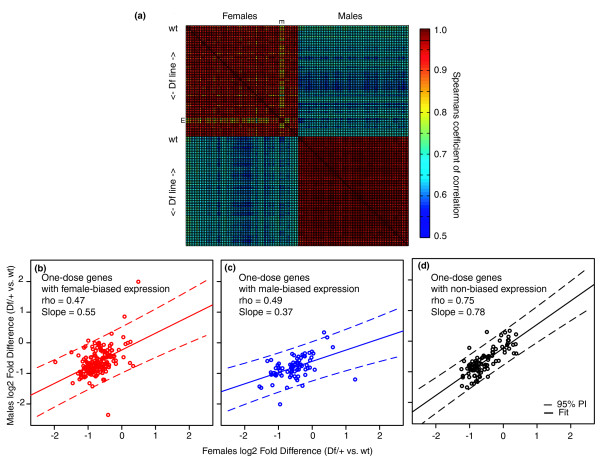
**Dosage compensation in the context of sex**. (a) Heat map showing overall correlations between each sample. The first three columns and rows in both the case of females and males represent expression profiles from the non-deleted *w^1118 ^*parental strain biological triplicates. The triplicates of the *Df(2L)ED793*/*+ *females are indicated ('E's). (b-d) Scatterplots of compensation ratios for the same genes when measured in females versus males. Wt, wild type.

At the genome-wide level, sex differences in gene expression were much more prevalent compared to the effect caused by gene dose. Expression profiles showed clear signatures of sex, and with the exception of *Df(2L)ED793*/*+ *females, only very subtle expression differences between lines within a sex (Figure 4a). The pervasive effect of sex on gene expression should drive the expression of one-dose genes to differing degrees in the sexes. If genes with sex-biased expression show different dosage compensation responses in females and males, this would suggest that compensation was network-dependent. To test this, we grouped genes detectably expressed in both sexes into those with female-, male-, or non-biased expression according to a database of sex-biased gene expression [33]. One-dose genes with female- or male-biased expression (Figure 4b,c), showed much less consistent compensation between the sexes (*ρ *= 0.47 and 0.49, respectively) compared to one-dose genes with non-biased expression (*ρ *= 0.75; Figure 4d), suggesting that network context modulated compensation.

### Expression of two-dose genes in *Df*/+ flies

Dosage effects and compensation by interactions within networks require perturbation detection by the network, which is then followed by feedback correction. In other words, perturbation propagation into the wild-type dose regions of the genome must precede compensation in temporal sequence. While all our observations were on steady-state conditions, we asked if there were any signatures of propagation in our datasets. Propagation would result in differentially expressed genes outside of the one-dose regions (two-dose genes, which includes dosage-compensated × chromosome genes in the case of males). Changes in two-dose gene expression were extensive. Collectively, the *Df*s we used altered the dose of approximately 5% of *Drosophila *genes, but we observed change in approximately 80% of *Drosophila *genes in at least one *Df*/*+ *line relative to the parental *w^1118 ^*line. Such changes did not appear to be a generalized response to aneuploid stress, as very few two-dose genes changed expression in all lines (nine in females, seven in males, and none in both sexes). Additionally, we observed no obvious ontology commonalities among these genes. The absence of a strong stress response [13] in our flies may be due to the rather limited number of genes with reduced gene dose in many of the deficiencies.

Our data showed that *Df*/*+ *genes compensated in the absence of a common two-dose genome response. The absence of evidence for induced expression (or repression) of a characteristic set of genes in the *Df*/*+ *flies is *sensu stricto *evidence against a general aneuploid response. We therefore used a reference composed of median expression values for all *Df*/*+ *lines to more cleanly examine two-dose gene expression change caused by particular *Df*/*+ *genotypes. Subtle expression change among two-dose genes was extensive and heterogeneous among the 21 lines (mean = 524.6 genes in females and 542.5 in males, or about 20 two-dose genes per one-dose gene; Additional file 4).

If changes in two-dose genome expression were due to regulatory interactions, then there should be a non-random set of changes that can be traced back to a causal one-dose gene. We tested for such gene expression network coherence and perturbation propagation by projecting our data onto the first sex-specific gene-expression network models for *Drosophila*. We constructed these networks from the expression data generated here, using mutual information, a quantity measuring the dependency between two variables, which has an important advantage over simple correlation methods, as it incorporates complicated nonlinear dependent relationships that better capture the relationships between complex genotypes and phenotypes [34] and relatedness in expression profiles [35]. Briefly, like many known biological networks, both our female and male networks (Additional files 5 and 6) exhibited scale-free properties; however, the power law exponents were different between sexes (-1.06 for female versus -1.35 for male), indicating that the overall structure of the networks differed between females and males (Table 2). Gene connectivity was higher in the female-specific network (*P *< 0.01 by degree-preserving edge shuffling; this method exchanges endpoints of edges under the restriction that the edges do not already exist in the network), and the subnetwork of genes with female-biased expression had significantly larger clustering coefficients than the subnetwork of genes with male-biased expression. Similarly, in the male network, genes with male-biased expression showed higher clustering than genes with female-biased expression. These differences in network structure were due only in part to sex-biased expression, as even among genes with non-sex-biased expression the clustering coefficients differed significantly in the female and male networks. Why the female and male mutual information models differ to this degree is not entirely clear, but for our purposes here, these two models provide distinct and independent frameworks for examining the propagation of dosage effects.

**Table 2:** Topological statistics for female and male mutual information networks

	Female network				Male network			
		
Expression bias	Female	Male	None	All	Female	Male	None	All
Number of nodes	3,456	789	1381	5,933	2,922	2,639	1,978	8,005
Clustering coefficient	0.44	0.30	0.36	0.44	0.12	0.31	0.19	0.23
Average neighbors	76.66	8.29	18.45	89.82	7.73	53.78	9.13	43.72
Density	0.022	0.01	0.013	0.015	0.003	0.02	0.005	0.005

**Figure 5:**
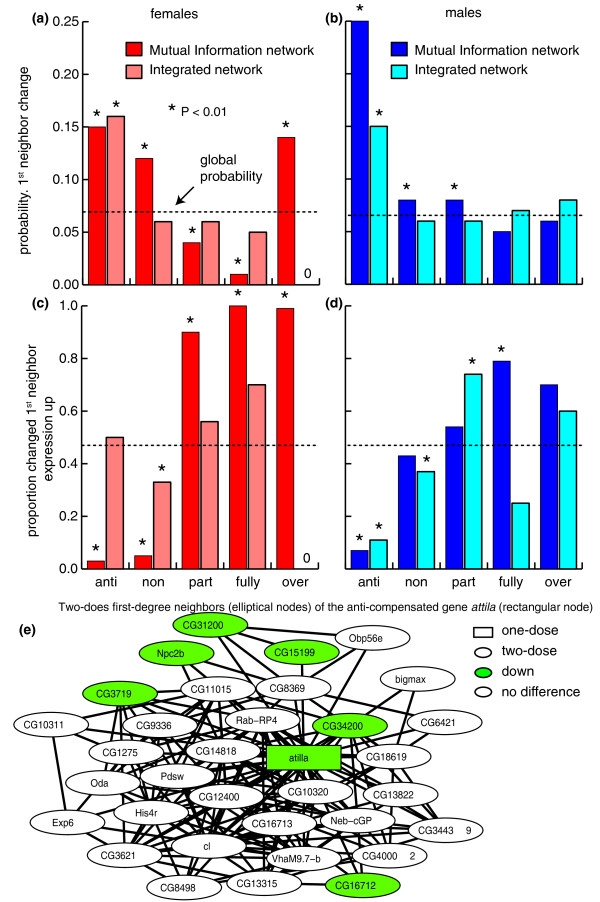
**Propagation of expression changes into two-dose genes**. (a,b) The probability of two-dose first-degree neighbors of one-dose genes changing expression by compensation response classification of the one-dose gene in females and males. (c,d) The proportions of two-dose first-degree genes with a positive direction of expression responses. Global averages for all two-dose genes are indicated (dotted lines). Significant differences from global values (*P *< 0.01, Chi-square test) are shown (asterisks). (e) Two-dose first-degree neighbors (elliptical nodes) of the anti-compensated gene *atilla *(rectangular node). Gene names are indicated for each node.

After projecting the expression data onto the network models we observed patterns of extensive connection between one-dose and two-dose genes. However, due to the large number of possible paths through these connections, we explored the relationship between one-dose genes and the local network, by examining expression changes for the unique first-degree two-dose neighbors of every one-dose gene in the sex-specific mutual information networks. Additionally, we also used a pre-existing network model that combines genetic interactions from *Drosophila *forward genetics, yeast two-hybrid data, and microarray expression datasets [36]. For all three networks, we calculated the probability of expression change in those unique first-degree neighbors among the different compensation categories (over-, partially, fully, non-, and anti-compensated). We found that one-dose genes with anti-compensated expression had two-dose first-degree neighbors with the highest probability of expression change (Figure 5a,b) irrespective of network model or sex. The propensity for first-degree neighbor change in expression networks centered on one-dose genes indicates that dosage effects are mediated, at least in part, by network interactions.

Outside of the anti-compensated class, the behavior of first-degree two-dose neighbors differed by sex and network. In females, there was significant stabilization of first-degree neighbor expression surrounding genes with partial or full compensation. These results are consistent with perturbation spreading, followed by robust resistance to expression change among neighbors of partially and fully compensated genes. In males, however, expression of first-degree neighbors approached the global average, with the notable exception of the anti-compensation class. The reason for this sex-difference is unclear.

Simple network interactions among first-degree neighbors centered on one-dose genes suggest that a given one-dose gene directly regulates some neighboring two-dose genes. Given that the one-dose gene is the cause of the perturbation, then we can determine whether propagation is due to positive or negative interactions in the gene pair. Globally, two-dose genes showed little bias in the direction of expression difference (Figure 5c,d). We therefore looked for skewing in the direction of two-dose gene responses among first-degree neighbors of one-dose genes. We observed a strong preference (*P *< 0.01, Chi-square test) for lower expression in the first-degree neighbors of anti- or non-compensated genes and for increased expression in the first-degree neighbors of partially, fully, and over-compensated genes (Figure 5c,d). An example of such a first-degree neighbor map centered on a one-dose gene is shown in Figure 5e. The non-random nature of first-degree neighbor change directionality strongly suggests that there was information flow between the one-dose genes and the surrounding two-dose genes. These relationships were dominated by sympathetic responses, suggesting positive regulation.

**Figure 6:**
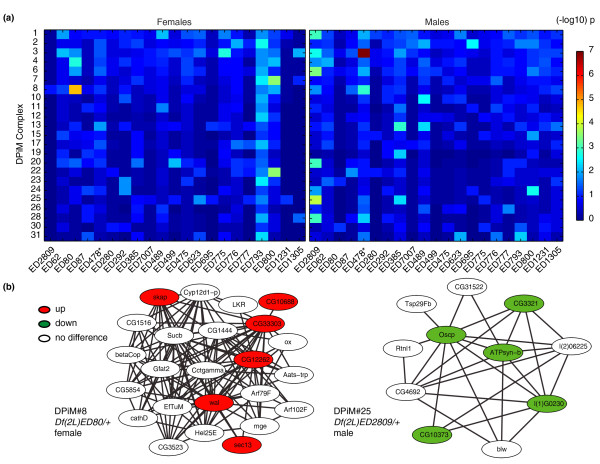
**Expression changes for two-dose genes encoding members of protein complexes**. (a) Heat map showing the joint probability of change in expression and uniformity in direction of change for DPiM protein complex models (rows) in each *Df*/*+ *line (columns) in females (left) and males (right). The double deletion *Df(2L)ED478*, *Df(2L)Hsp60c *is indicated (asterisk). The inverse *P *(hypergeometric test) log scale is shown. (b) Two examples showing the expression of two-dose genes encoding two different complexes in the indicated *Df*/*+ *line and sex. Increased (red), decreased (green) and no expression difference (open) are indicated. Gene names are indicated for each node.

There was changed expression of two-dose genes beyond what we could unambiguously trace through the networks. We asked if these changes in expression were coherent by focusing on genes encoding members of protein complexes. The *Drosophila *Protein interaction Map (DPiM) is a *Drosophila *protein-protein interaction model for protein complexes based on co-affinity purification followed by mass spectrometry [37]. We examined expression changes in the *Df*/*+ *lines for two-copy genes encoding members of 23 high-confidence multi-subunit complex models from DPiM to determine if changed expression in one member was associated with an enriched chance for expression change in another gene encoding a complex member (Figure 6). Of the 966 cells in the matrix (23 complexes × 21 lines), we observed significant co-expression change in 37 cases (*P *< 0.01, hypergeometric test). These data suggest that expression changes in the one-dose region of the genome preferentially affect two-dose genes encoding members of the same protein complexes. This is strong evidence that one-copy genes result in coherent expression perturbation in the two-dose genome beyond the first-degree neighbors.

## Discussion

The relationship between DNA dose and gene expression in *Drosophila *is poorly understood, but gene product balance is clearly important [7,38]. For example, assembly of multi-subunit complexes such as ribosomes is highly sensitive to changes in the dose of constituent proteins. Additive effects of massive gene dose deviations are incompatible with life in *Drosophila *and, given a sensitive assay, the gene doses of single modifiers have pronounced phenotypic effects. It follows that there should be a response to gene dose beyond the genes with reduced dose and some of those changes will involve feedback onto the genes with altered dose. Studies of *Drosophila *structural variants have shown partial dosage compensation of autosomal genes as measured as a population of genes with altered dose [8-10,14]. This partial compensation could be the result of uniform compensation of all genes, or heterogeneous responses with a characteristic mean.

### Network properties contribute to dosage compensation

Briefly, we have shown that one-dose genes show individual expression responses to reduction in dose. While we cannot rule out some contribution of a stereotypic aneuploid response resulting in partial compensation, the overall response is highly heterogeneous. In humans, it also appears that individual genes can show very good (rare), or negligible (common), compensation, suggesting that gene-by-gene compensation is not restricted to *Drosophila *[39]. Inverse effects, where gene expression is anti-correlated with gene dose, are well-known in maize [40], and we see rare over-compensation resulting in higher expression when dose is reduced in *Drosophila*. We suggest that the compensation of one-dose genes occurs in the context of the surrounding gene expression network. Similar effects occur in mice, where some one-dose genes show compensation during specific temporal windows, strongly suggesting feedback regulation [41]. We also demonstrate that perturbation coherently propagates from one-dose genes into the network. This indicates that autosomal dosage compensation and the consequences of the absence of dosage compensation are, at least in part, network properties. The differences in compensation in these diverse systems may be due to the relative portions of compensation gene classes, and/or network architecture, rather than a gross difference in gene behavior between organisms.

We showed that the gene-by-gene response to dose depends on two related factors; gene expression level and network context. Compensation is poorer for highly expressed genes, and indeed the relationship between compensation and expression shows some hints of converging on non-compensation at high expression levels. Our results are consistent with the observation that tissue-specifically expressed genes were better compensated in *Drosophila *compared to ubiquitously expressed genes [8], as non-ubiquitously expressed genes show lower expression in whole animal samples. Additionally, our data at high expression levels is consistent with the response in yeast, where highly expressed genes show no dosage compensation at the protein level while a minority are perfectly compensated [42]. Our results differ from another recent report that highly expressed *Drosophila *genes were better compensated [14]. Because of the highly heterogeneous gene-specific response to dose, it is quite possible that these differences in conclusions are due to the particular regions of the genome examined. While we do not understand why compensation depends on steady-state expression level, it is possible that better compensation of poorly expressed genes is due to robust control of expression where low abundance increases deviations due to stochastic noise. As an extreme example, a transcript present at, or below, one copy per cell must result in wild swings in fold relationships to other transcripts, and might be an excellent candidate for compensation. Poor compensation of highly expressed genes may be due to 'speed limits' imposed by maximal rates of transcription for a particular arrangement of regulatory sequences at that locus.

Our study demonstrates that there are coherent patterns of expression change in potentially co-regulated complexes and immediate neighbors of one-dose genes. It seems likely that both kinetics and active regulation are components of network mediated dosage compensation and propagation. Transcription is an enzymatic process, and changes in enzyme concentration in pathways are buffered by substrate and reactant concentrations that depend on other enzymes in the pathway [20], such that flux varies between no change and change of the same magnitude as the dose change. However, buffering does not explain the anti- and over-compensation we observed, suggesting that active regulation via feedback is also a component of dosage compensation. Buffering and feedback are not mutually exclusive. For example, the yeast galactose network (involving GAL2, GAL3, GAL4 and GAL80) is robust to gene dose alterations through a simple two-component system with at least one inhibitor and one activator regulating the pathway. However, activator-inhibitor interactions and stoichiometry in the galactose network have profound effects on the robustness of the network [43].

Our work suggests that the anti-compensated genes might result in the most damage to the rest of the gene expression network, or minimally, that the damage is more easily traced into the expression network in our models. These dose effects indicate that anti-compensated genes are weakly haploinsufficient and are good candidates for pathological variants. At least in females, the over-compensated genes also appear to result in clear propagation of perturbation and are likely to be damaging to the expression network. Another female-restricted response suggests that genes with partial or full dosage compensation increase the robustness of the local expression network centered on those genes. That males show different propagation patterns could be due to inherent differences between females and males or differences in network model quality. Despite these differences, in both female and males we observed sympathetic changes in expression of first-degree neighbors of one-dose genes. These data suggest that most causal relationships identified are positive, despite the fact that mutual information networks identify both correlated and anti-correlated relationships.

### What about sex chromosomes?

While we do not examine × chromosome dosage compensation in this manuscript, our findings do have some implications for some of the models for sex chromosome dosage compensation. The majority of studies suggest that the male × chromosome is upregulated to achieve compensation in *Drosophila *[15]. However, it has also been suggested that interaction between the autosomes and the × chromosome contributes to × chromosome dosage compensation by lowering autosomal expression in males [38]. The relationship between the non-compensated genes and first-degree neighbor expression we observe here is sympathetic. If this is also true for × chromosome genes, then one effect of non-compensation of X-linked genes would be to lower expression of first degree neighbors encoded on autosomes. Therefore, × chromosome-autosome interactions might act to partially balance gene expression by lowering autosomal expression. While we have previously reported that models calling for up-regulation of the × in *Drosophila *males explains more of dosage compensation than possible network interactions with the autosomes [10], it is quite possible that such interactions exist. It is perhaps even more likely that these interactions existed and shaped dosage responses during the evolution of the × and Y chromosomes from an ancestral autosome pair [44]. As genes are lost from neo-Y chromosomes there is a gradual crisis that is not effectively controlled by chromosome-wide mechanisms until Y-chromosome gene loss is extreme (evolutionarily premature dosage compensation would make males functionally triploid for genes present on the × and Y). Sex chromosome-wide mechanisms that have evolved also create imbalances. Network interactions between the × and autosomes could also contribute to the equilibration of × and autosome expression in XY male mammals and in XX females following × inactivation [18]. Similarly, network effects might also help explain dosage compensation in the absence of MSL in the early *Drosophila *XY male soma and mitotic germline [9,17,18].

Our results also point out the complications of characterizing sex chromosome dosage compensation in the absence of a baseline value for autosomal compensation. For example, in light of our findings, it is unclear if partial sex chromosome compensation in birds [45] is due to a generic response to monosomy or a chromosome-specific compensation mechanism with limited efficacy. On the other hand, possible sex chromosome heterogeneity in baseline compensation in the absence of a chromosome-wide mechanism also cautions against using global expression values to make broad statements about sex chromosome dosage compensation. Specifically, it has been proposed that intermediate × chromosome compensation in the wild-type *Drosophila *male germline is due to a fixed fold effect of failed × chromosome dosage compensation [46], rather than complications due to measuring expression in mixes of cells showing dosage compensation, sex-biased gene content, and the precocious × chromosome inactivation that occurs in male germ cells ranging from *Caenorhabditis elegans *to human [18,47]. The clearest conclusion for the study of sex chromosome compensation is that one should not assume that the two-fold difference in gene dose is easily corrected by a fixed-fold dosage compensation system, as the baseline expression for 'non-compensated' sex chromosome genes may well differ among sex-linked genes. The study of sex chromosome dosage compensation will need to be coupled with studies of dosage compensation elsewhere in the genome.

### Building better network models

Our network modeling shows a common thematic connection between one-dose genes and the rest of the genome, but these models are far from complete and differ, for example, in the specific genes we called first-degree neighbors. Systematic subtle perturbation using gene dose is a good tool for generating better network models. Specifically, since we can trace propagating changes in engineered *Drosophila *where the causal dose change is known, we can move beyond connectivity to information flow within current network models and use these data iteratively to build better models. For example, the expression values for a given gene pair connected by an edge are the result of one gene regulating the other (directly or indirectly) or both genes being co-regulated by a common first-degree neighbor. In a positive interaction, the predicted response to an instantaneous gene dose reduction is reduced expression of directly regulated neighbors; however, if a third gene responds to one-dose expression by increasing the expression of this co-regulated pair, then the first degree neighbor of the one-dose gene should be over-expressed. Indeed, we observed that partially compensated genes were enriched for over-expressed first-degree neighbors in both sexes. A larger data set, where each node in a subnetwork is one-dose in one experiment and two-dose in the others, should allow us to unambiguously determine if relationships are directional and, if so, whether the effect is positive or negative. With better models, we should be able to predict information flow, and perhaps dose-dependent genetic interactions resulting in oligogenic phenotypes. Finally, if we can establish a basic understanding of gene dose responses in *Drosophila*, we may be able to apply basic rules to copy number variations associated with human disease, which also appear to be mediated by network responses [48].

## Materials and methods

### Flies and samples

We obtained flies from the *Drosophila *stock center (Bloomington, IN, USA). We crossed DrosDel males to virgin *w^1118 ^*females to remove balancer chromosomes. We determined that the line initially labeled *Df(2L)ED748 *had the breakpoints reported for *Df(2L)ED478*, and changed the labeling in this report accordingly. This line also carried an additional 2L deletion (Figure 1). Flies were grown under constant temperature and humidity (25°C; 60% relative humidity) on San Diego Stock Center cornmeal media [49]. We pooled 50 to 60 sexed adults (5 days post-eclosion) for RNA extraction for each of 3 to 4 replicate preparations. Total RNA was extracted using TRIzol® (Invitrogen, Carlsbad, CA, USA) and poly A+ mRNA was enriched using Oligotex (Qiagen, Valencia, CA, USA) following the manufacturer's instructions. mRNA quality was scored by the presence of tight rRNA bands in Bioanalyzer profiles (Agilent, Santa Clara, CA, USA). We extracted DNA using the LiCl method [50] and quantified on a Nanodrop (Thermo Fisher, Wilmington, DE, USA).

### Arrays and sequencing

All array and sequence data are available from the Gene Expression Omnibus (GEO) [51]. See GEO GPL8593 for array platform details and GEO GSE31407 for complete methods and supplemental information. We used a 12-plex 60-mer probe microarray, 080523_D_melanogaster_5.5_expr (Roche Nimblegen, Madison, WI, USA), and performed experiments in at least biological triplicates as described [10] in a chamber with air passed through NoZone ozone scrubbers (SciGene, Sunnyvale, CA, USA). All array data were in log_2 _scale. We normalized all microarray data triplicates with rank correlation ≥0.8 using Robust Multi-Chip Averaging [52] to produce a gene level metric of expression. Two sample hybridizations failed to meet this threshold and were not further considered. We then set the threshold of detected expression at 2 SD above mean hybridization intensity to control probes. We demanded that a given gene show within-sex expression above this threshold in all tested lines. We used two types of references in the manuscript. When testing for a global effect of aneuploidy, we used median expression of the *w^1118 ^*line as a denominator. When we were testing for the effect of particular deficiencies, expression ratios were comparisons to a composite reference built from the median expression values from all experiments. Expression differences were called by false discovery rate-corrected (*P *< 0.05) moderated *t-*tests [53,54].

For RNA-Seq, 100 to 200 ng of poly-A+ mRNA from samples along with external spike-in control libraries were prepared for sequencing on a GAII (Illumina, San Diego, CA, USA) or SOLiD 4 (Life Technologies, Carlsbad, CA, USA). We used 8 [55] external control RNAs for Illumina RNA-Seq and 96 [31] ERCC external control RNAs for SOLiD RNA-Seq. For the ERCC controls mix 1:mix 2 ratios contain three subsets of 32 RNAs at 1:1, 1.5:1, and 1:1.5, with a dynamic concentration range of 2^20^. Mix 1 was added to wild-type mRNA and mix 2 was added to *Df*/*+ *mRNAs. For Illumina runs, we used 36 bp reads that passed default parameters, Chastity ≥0.6 (Illumina). For SOLiD runs, we used only the forward read and trimmed these reads from 50 to 36 bp based on analysis of read quality and to make data comparable to Illumina data. For DNA-Seq, 5 μg of DNA was prepared as described [10] and sequenced on a GAII or HiSeq 2000 (Illumina) as outlined for RNA-Seq.

We used the dm3 *Drosophila melanogaster *sequence build [56] from the UCSC Genome Browser [57] as a reference (excluding Uextra) for alignment using Bowtie v.0.12.7 settings -v 2 -m 1 [58] and FlyBase r5.29 for annotations [59]. We quantified expression using HTSeq union mode [60], and used the unique mapping reads to calculate reads per kb per million mapped (RPKM) as the normalized metric of gene expression. We identified the novel *Df *breakpoint with rSW-Seq [61] and determined fold-difference for aneuploid segments with samtools [62], which we expressed as reads per million (RPM). All but the smallest 5 kb deletion was detected using this method. We did not attempt to measure single nucleotide polymorphisms. We visualized expression data with Bioconductor tools [63] or MatLab (Mathworks, Natick, MA, USA), and DNA-Seq coverage with Bedtools [64] and the UCSC Browser.

### Network analysis

We used a two-step procedure to classify the expression of one-dose genes into five groups: anti-compensated, non-compensated, fully compensated, partially compensated, and over-compensated. First, using a moderated *t*-test, we tested the null hypothesis that the expression of one-copy genes was reduced by half compared to the DrosDel reference values for each gene. We rejected the null hypothesis for all genes with *P *< 0.05 (limma package from Bioconductor [63] with false discovery rate by Benjamini-Hochberg correction [54]). The genes for which the null hypothesis was not rejected were classified as non-compensated. We classified genes for which the null hypothesis was rejected and expression was lower than the expected two-fold reduction as anti-compensated. Next, the genes for which the null hypothesis was rejected and the expression was higher than the expected two-fold reduction (199 genes in females and 242 gene in males) were stratified into compensation levels - fully compensated, partially compensated and over-compensated - using cutoff values defined as follows. To set appropriate cutoff values, we first estimated the distribution of log fold change in this group by sampling 1,000 times with repetition, and subsequently computed normal distribution based cutoff for the quintiles 2.5% and 97.5%. These cutoffs where then adjusted by subtracting the sampled population mean, thus centering the confidence interval at 'no change' relative to the reference. Finally, the genes with mean log-fold expression change between the cutoffs were classified as fully compensated, the ones above the upper cutoff as over-compensated and ones below the lower cutoff as partially compensated.

We used our gene expression data (subtracted mean expression for each gene across lines and replicates/SD) as a variable and estimated mutual information (MI) for all possible pairs of genes by a kernel method [65] to construct the sex-specific models (kernel width = 0.3; edges with MI *P *< 0.005). Unlike simple correlation, MI tests non-linear relationships and does not require that the distribution of variables is normal. In addition, MI networks have been shown to perform well on simulated data and to be more resilient to estimation errors [66]. We used other networks as described by the creators [36,37]. Twenty-three protein complexes enriched for ontology terms (*P *< 0.005) and having ten or more members were selected directly from the DPiM network without further processing [36,37], and we used the hypergeometric test for significance of expression change.

We visualized networks in Cytoscape v.2.8 and used the Network Analysis and Random Network plug-ins to fit power-law models, generate randomized networks and generate descriptive statistics for the female and male networks [67,68]. We used the one-versus-everyone approach [69] to identify significantly changed 1st degree neighbor expression change, and we calculated the global probability of changed expression from all nodes in each network and compared to observed by Chi-square test. Probabilities are indicated in the main text. We performed network statistics and analysis in MatLab (Mathworks).

## Abbreviations

*2L*: left arm of chromosome 2; bp: base pair; *Df*: Deficiency; DPiM: *Drosophila *Protein interaction Map; DrosDel: European *Drosophila *deletion collection ERCC: External RNA Controls Consortium; GEO: Gene Expression Omnibus; MI: mutual information; MSL: male specific lethal; RPM: reads per million; SD: standard deviation.

## Competing interests

The authors declare that they have no competing interests.

## Authors' contributions

JHM, DYC, NRM, JA, TMP, and BO conceived and directed the project. JHM, DYC, JM, SM, and HES acquired data. JHM, DYC, SM, RD, MS, CGA, LJ, JA, TMP, and BO analyzed results. JHM, DYC, TMP, and BO wrote the manuscript. All authors have read and approved the manuscript for publication.

## Supplementary Material

Additional file 1 **Figure. DNA-seq of *Df*/*+ *lines**. Click here for file

Additional file 2 **Expression of one-dose genes (Excel)**. Click here for file

Additional file 3 **Compensation by position along chromosome arm 2L**. Click here for file

Additional file 4 **Expression of two-dose genes (Excel)**. Click here for file

Additional file 5 **Female mutual information network (Cytoscape)**. Click here for file

Additional file 6 **Male mutual information network (Cytoscape)**. Click here for file
