# Supplementary material for: Mediation of Drosophila autosomal dosage effects and compensation by network interactions
Source: Genome Biol. 2012 Apr 24;13(4):R28. doi: 10.1186/gb-2012-13-4-r28 (PMC3446302; doi:10.1186/gb-2012-13-4-r28)
Supplement: Additional file 1 — Figure. DNA-seq of Df/+ lines. [file gb-2012-13-4-r28-S1.PDF]

DNA-Seq Coverage on Chromosome 2L

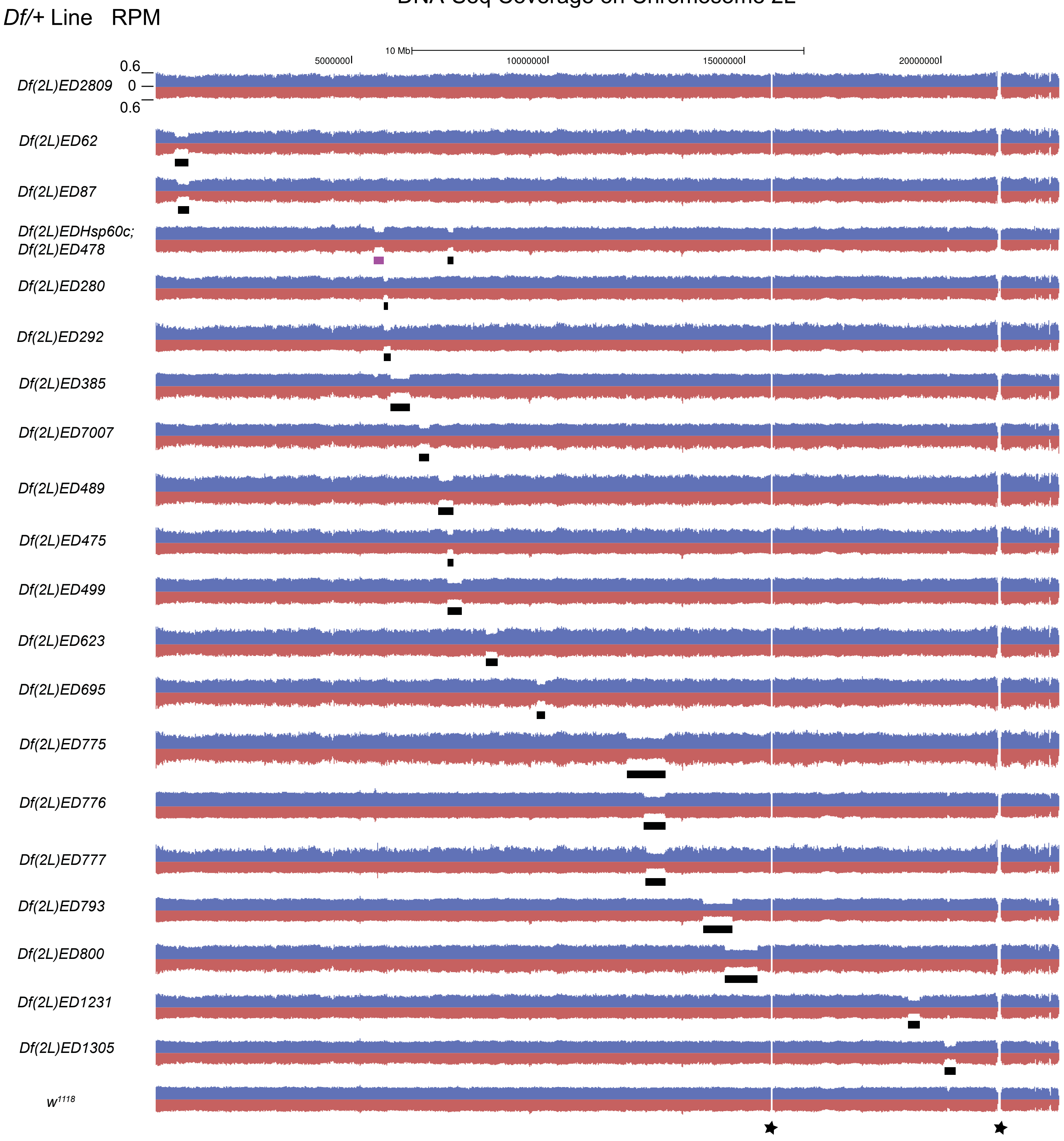

- Coverage in males
- Coverage in females
- DrosDel
- Novel deficiency
- ★ No Unique Alignment (repeats)

DNA-Seq seq density coverage values of reads from libraries made from female (red) or male (blue)flies heterozygous for the indicated *Dfs*. The data from the sexes are presented as mirror image plots. The units of measure are reads per million mapped reads (RPM). Only data for Chromosome arm 2L are shown. The smoothing is the result of UCSC browser scaling of uploaded .wig files. The locations of deletions are shown (rectangles, also see key to the left). Regions where repeats prevented unique mapping result in coverage gaps unrelated to any structural rearrangments in the flies (stars).
