# Supplementary material for: Mediation of Drosophila autosomal dosage effects and compensation by network interactions
Source: Genome Biol. 2012 Apr 24;13(4):R28. doi: 10.1186/gb-2012-13-4-r28 (PMC3446302; doi:10.1186/gb-2012-13-4-r28)
Supplement: Additional file 3 — Compensation by position along chromosome arm 2L. [file gb-2012-13-4-r28-S3.PDF]

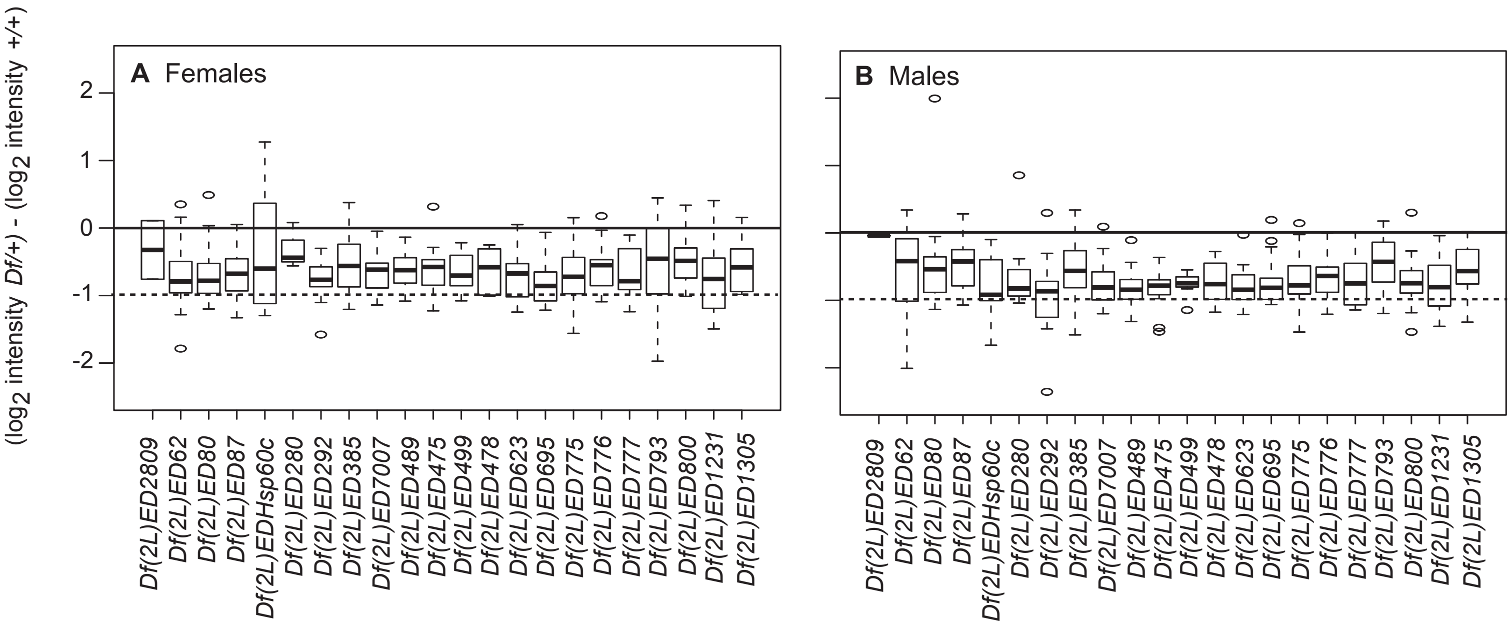

Box and whisker plots of expression ratios of genes present at one-dose in the indicated deficiencies and two-dose in wildtype females (A) and males (B). Median (bold line), 25th-75th quartiles (box), 1.5X interquartile range (whiskers), and outliers (circles) are shown. Deficiencies are listed in telomere to centromere order along chromosome 2L. Expected values for fully-compensated genes (solid line) and non-compensated genes (dashed line) are shown.
